# Risk of de novo proteinuria following hospitalization with acute kidney injury

**DOI:** 10.1186/s12882-023-03209-y

**Published:** 2023-06-15

**Authors:** Saniya S. Bonde, Warda Zaman, Raphael Cuomo, Rakesh Malhotra, Etienne Macedo

**Affiliations:** 1 Department of Medicine, University of California San Diego, San Diego, CA USA; 2 East Bay Nephrology Medical Group, Berkeley, CA USA; 3 Department of Anesthesiology, School of Medicine, University of California San Diego, San Diego, CA USA; 4 Division of Nephrology and Hypertension, Department of Medicine, University of California San Diego, La Jolla, CA USA

**Keywords:** Proteinuria, Acute kidney injury

## Abstract

**Background:**

Acute Kidney Injury (AKI) incidence has continued to rise and is recognized as a major risk factor for kidney disease progression and cardiovascular complications. Early recognition of factors associated with post-AKI complications is fundamental to stratifying patients that could benefit from closer follow-up and management after an episode of AKI. Recent studies have shown that proteinuria is a prevalent sequela after AKI and a strong predictor of complications post-AKI. This study aims to evaluate the frequency and timing of the development of de-novo proteinuria after an AKI episode in patients with known kidney function and no prior history of proteinuria.

**Methods:**

We retrospectively analyzed data from adult AKI patients with pre- and post-kidney function information between Jan 2014 and March 2019. The presence of proteinuria determined before and after index AKI encounter was based on ICD-10 code and/or urine dipstick and UPCR during the follow-up period.

**Results:**

Of 9697 admissions with AKI diagnoses between Jan 2014 and March 2019, 2120 eligible patients with at least one assessment of Scr and proteinuria before AKI index admission were included in the analysis. The median age was 64 (IQR 54–75) years, and 57% were male. 58% (n-1712) patients had stage 1 AKI, 19% (n = 567) stage 2 AKI, and 22% (n = 650) developed stage 3 AKI. De novo proteinúria was found in 62% (n = 472) of patients and was already present by 90 days post-AKI in 59% (209/354). After adjusting for age and comorbidities, severe AKI (stage 2/3 AKI) and diabetes, were independently associated with increased risk for De novo proteinuria.

**Conclusion:**

Severe AKI is an independent risk factor for subsequent de novo proteinuria post-hospitalization. Further prospective studies are needed to determine whether strategies to detect AKI patients at risk of proteinuria and early therapeutics to modify proteinuria can delay the progression of kidney disease.

**Supplementary Information:**

The online version contains supplementary material available at 10.1186/s12882-023-03209-y.

## Introduction

The long-term effect of acute kidney injury (AKI) in CKD progression and cardiovascular complications has been recognized over the last decade [1, 2]. The burden of AKI is expected to increase as studies using a consistent AKI definition have shown an increasing incidence, 14% per year [3]. Furthermore, hospital readmissions and recurrence of kidney injury are also frequent following an episode of AKI [4–6]. Acute kidney injury has also long-term effects beyond the kidneys, and follow up after an AKI episode can pottentialy improve patient outcomes. Currently, non-recovery of kidney function and preexisting comorbidities are the main factors determining outpatient follow-up after an episode of AKI in hospitalized patients. Assessing the development of proteinuria as a sequela of AKI can help stratify patients at increased risk of kidney and cardiovascular complications and may help understand how AKI accelerates the progression of CKD.

Several cohort studies have confirmed that non-recovery of kidney function following an episode of AKI is a major contributor to the prevalence of chronic kidney disease (CKD) [7–12]. The growing evidence establishing short- and long-term consequences of AKI has emphasized the need for improvement in the management and follow-up of patients with AKI [13]. Some studies have suggested that nephrology follow-up can be beneficial [14–17]. However, given the growing number of patients discharged with partial recovery of kidney function after an AKI episode, selecting patients with a higher risk of complications and tailoring post-AKI care to the specific needs of different patients or patient subgroups may be required [18].

In CKD, de novo proteinuria can significantly impact disease progression and patient prognosis. Proteinuria can damage the kidneys by promoting inflammation and oxidative stress, leading to fibrosis and scarring. The extent of proteinuria is considered one of the most important predictors of CKD progression. Higher proteinuria is associated with a faster decline in kidney function and a higher risk of developing end-stage renal disease (ESRD) [9].

With the rising incidence of AKI, identifying patients at higher risk for complications is essential. The main risk factors for non-recovery from AKI include baseline eGFR, increased age, diabetes, proteinuria, and duration and severity of AKI [7, 8, 10, 12, 19–22]. Based on serum creatinine (Scr) levels, even patients with presumed complete recovery of kidney function have a higher risk of CKD progression, cardiovascular events, and mortality [7, 23]. Analysis of patients with complications following an AKI episode suggests that the need for follow-up should not be determined solely on the changes in estimated glomerular filtration rate (eGFR) but also on developing other complications such as de novo proteinuria, a marker of kidney damage. In this study, we aim to identify the risk of de-novo proteinuria in patients following an episode of in-hospital AKI.

## Methods

We extracted electronic medical record (EMR) information from patients 18 years or older with AKI diagnosis based on ICD 9/10 codes during hospital admission (index encounter) between January 1st, 2014, and March 3rd, 2019, at the UC San Diego Health System. Approval to perform anonymous analyses of routinely collected clinical data was obtained with a waiver of informed consent from the Institutional Review Board of the University of California, San Diego. We included patients with at least one pre-AKI (pre-index encounter) and one post-AKI (post-index) encounter. We excluded patients with a kidney transplant or dialysis history prior to AKI. An encounter was defined as any inpatient or outpatient hospital visit in which kidney function was assessed. We extracted labs and vital signs from encounters up to one year before the index encounter.

All the comorbidities were determined through ICD-10 codes present before and at the index encounter. The specific codes for these can be found in Supplemental Table 1. Baseline eGFR is the mean value between 0 and 90 days before the AKI admission. If no data were available in this period, we considered baseline eGFR the closest value within 365 days of the admission day.

Proteinuria outcome was assessed based on the presence or absence of the ICD-10 code and/or laboratory information during follow-up after the index encounter. The absence of data was not considered an absence of outcome but as missing data and therefore removed from the analyses. Based on laboratory information, proteinuria was considered present if urinalysis (UA) showed 1 + to 4+, or urine protein creatinine ratio (UPCR) was greater than or equal to 0.3. Proteinuria was considered absent if UA results were negative, trace or invalid, and/or UPCR < 0.3. Baseline proteinuria status was based on the information from pre-index encounters, and information from the index encounter was not used to define proteinuria outcome. We further evaluated the timing of the development of proteinuria based on intervals: 0–90 days, 91–180 days, 181–365 days, 366–540 days, 541–730 days, and > 730 days post-index day.

Continuous variables were expressed as the mean (standard deviation (SD)) or median (interquartile range [IQR]) and analyzed by unpaired t-test or the Wilcoxon rank sum test, as appropriate. Categorical variables are expressed as absolute (n) and relative (%) frequency.

We performed a multivariate logistic analysis to evaluate risk factors for developing de-novo proteinuria after an AKI episode. A p-value < 0.05 was considered statistically significant. Statistical analysis was performed via IBM SPSS Statistics, Version 27.0. The Hosmer-Lemeshow test for goodness of fit was used to evaluate the models.

## Results

**Fig. 1:**
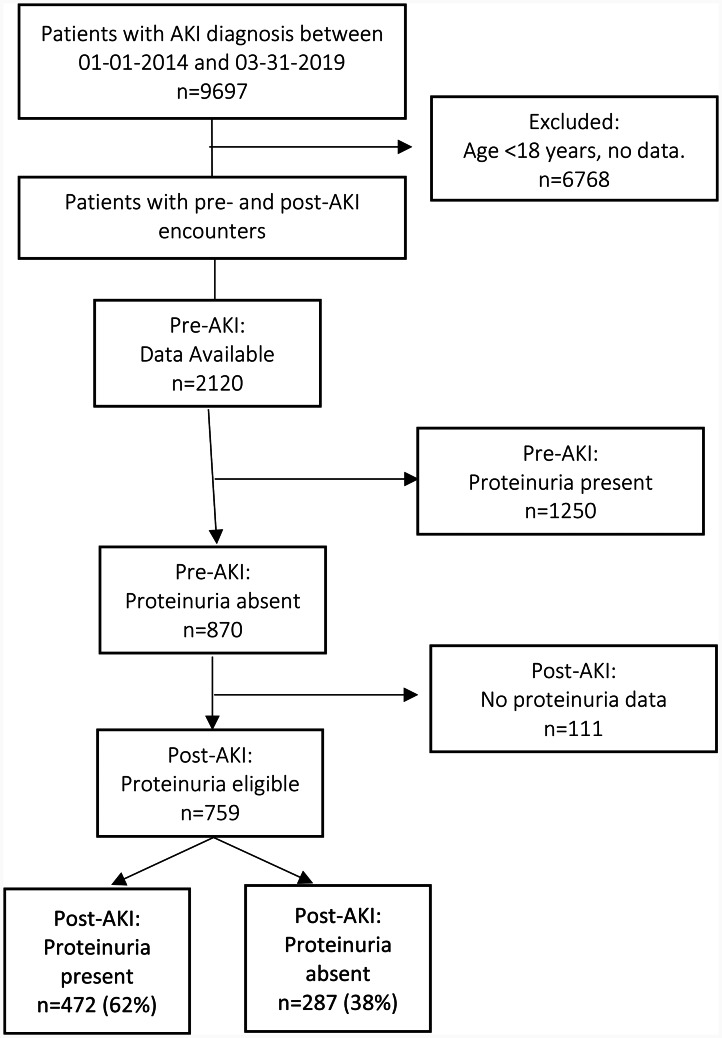
CONSORT diagram

Between 2014 and 2019, we had 9697 admissions with AKI diagnoses. There were 2120 eligible patients with at least one assessment of Scr and proteinuria before AKI index admission. Figure 1 presents the Consolidated Standards of Reporting Trials (CONSORT) diagram and demonstrates patient eligibility based on the presence or absence of proteinuria at the pre-index encounter. Patients who were proteinuria-absent at index encounter and had data available at post-AKI were considered proteinuria-eligible or at risk of developing this outcome post-AKI.

### Baseline characteristics

Patient baseline demographic information is shown in Table 1. The median age was 64 (IQR 54–75) years, and 57% were male. The prevalence of hypertension was 64% (n = 1877), and 32% (n = 946) had diabetes. The median baseline eGFR was 60 (IQR, 45–77) mL/min/1.73m2. Stage 1 AKI was found in 58% (n-1712) patients, 19% (n-567) developed stage 2 AKI, and 22% (n = 650) developed stage 3 AKI during admission.

**Table 1:** Patient characteristics at hospital index admission

	All patients N = 2929	Did not develop proteinuria N = 287	Developed proteinuria N = 472	p value
Demographics				
Age, yr, median (IQR)	64 (54–75)	65 (55–76)	65 (55–78)	0.79
Sex, n (%)				0.13
Female	1264 (43)	131 (46)	242 (52)	
Male	1665 (57)	156 (55)	230 (49)	
Race, n (%)				0.71
White	1745 (60)	174 (61)	276 (58)	
Black/African American	418 (14)	34 (12)	57 (12)	
BMI, kg/m^2^, median (IQR)	26 (23–31)	26 (24–31)	25.8 (22–31)	0.43
Comorbidities, n (%)				
Diabetes	946 (32)	76 (27)	160 (34)	0.03
Congestive heart disease	819 (28)	69 (24)	130 (28)	0.29
Coronary artery disease	733 (25)	72 (25)	102 (22)	0.27
Myocardial infarction	175 (6)	10 (4)	24 (5)	0.30
Cerebrovascular disease	470 (16)	48 (17)	84 (18)	0.71
Peripheral vascular disease	253 (9)	27 (9)	51 (11)	0.54
Liver disease	499 (17)	56 (20)	90 (19)	0.88
Dyslipidemia	1200 (41)	128 (45)	203 (43)	0.67
Hypertension	1877 (64)	176 (61)	321 (68)	0.06
AKI KDIGO Stages, n (%)				< 0.001
AKI Stage 1	1712 (58)	196 (68)	247 (52)	
AKI Stage 2	567 (19)	44 (15)	106 (23)	
AKI Stage 3	650 (22)	47 (16)	119 (25)	
Labs, median (IQR)				
Admission SCr (mg/dl)	1.4 (1.2-2.0)	1.4 (1.1–1.8)	1.4 (1.1–1.9)	0.47
Admission eGFR (ml/min/1.73m^2^)	45 (30–63)	47 (33–65)	46 (30–64)	0.57
Baseline eGFR (ml/min/1.73m^2^)*	60 (45–77)	61 (50–75)	53 (42–71)	0.008
Admission albumin (g/dl)	3.7 (3.2–4.1)	3.8 (3.3–4.2)	3.7 (3.3–4.1)	0.03

* Baseline eGFR is mean of eGFRs retrieved from 0–90 days before the AKI admission. If no data were available, baseline eGFR reflects the eGFR from the day closest to and within 365 days of the admission day. There were 90 patients who did not develop proteinuria and had a baseline eGFR, while there were 114 patients who developed proteinuria and also had a baseline eGFR

Abbreviations: IQR, interquartile range; BMI, body mass index; AKI, acute kidney injury; KDIGO, Kidney Disease Improving Global Outcomes; Scr, serum creatinine; eGFR, estimated glomerular filtration rate

**Fig. 2:**
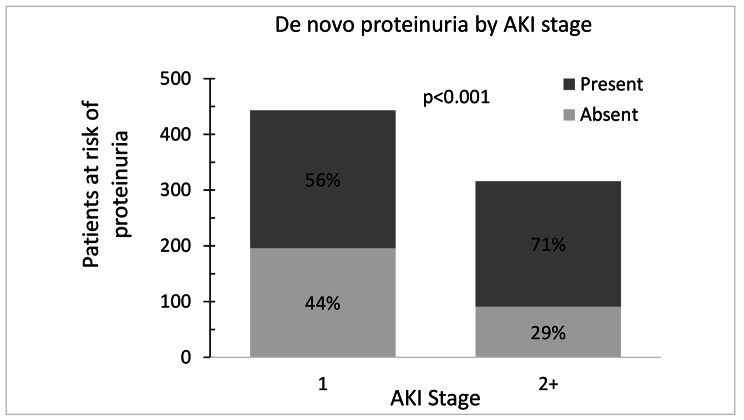
Patients at risk of de novo proteinuria stratified by AKI staging

Out of 2120 patients with laboratory data available before AKI hospitalization, 1250 patients had proteinuria before hospitalization with AKI and 870 patients without proteinuria. At post-AKI encounter, 759 patients available data on proteinuria and thus were considered proteinuria-eligible and included in the analysis. About 62% (n = 472) of patients developed de novo proteinuria. Figure 2 demonstrates the incidence of de-novo proteinuria, which was higher in patients with severe AKI (stage 2/3 AKI) as compared to patients with stage 1 AKI (71% (225/316) vs. 56% (247/443); p < 0.001). Supplemental Table 2 shows urinalysis data for defining proteinuria. Roughly 42% (n = 202) patients had 1 + proteinuria, whereas 45% (n = 211) patients had 2 + proteinuria on the urine dipstick.

Table 2 demonstrates the timing of the development of de-novo proteinuria since the index hospitalization. By day 90 post-AKI, 209/354 patients (59%) had already developed de novo proteinuria.

**Table 2:** First evidence of de novo proteinuria by time intervals

First evidence of proteinuria after index admission (d)	Presence of proteinuria after index admission (d)						Total
	0–90	91–180	181–365	366–540	541–730	> 730	
0–90	209	61	53	41	29	37	
91–180		63	19	16	10	10	
181–365			82	12	12	20	
366–540				44	13	19	
541–730					27	12	
> 730						47	
Proteinuria -eligible, n	354	107	126	66	38	68	759
Proteinuria-present by first time, n (%)	209 (59)	63 (59)	82 (65)	44 (67)	27 (71)	47 (69)	472

Legend: The columns represent the time interval to detection of proteinuria, and rows indicates number of patients with proteinuria detected for the first time. To calculate the percent with first evidence of de novo proteinuria, the number of patients who demonstrated proteinuria was divided by the number of patients who had been proteinuria-eligible at that same time interval

In an unadjusted model, patients with severe AKI (stage 2/3 AKI) had significantly increased odds of developing de novo proteinuria compared to patients with stage 1 AKI (OR = 1.96; 95% CI 1.44 to 2.67). This difference remained statistically significant after adjusting for age, sex, ethnicity, and presence of chronic comorbidities (OR = 1.95; 95% CI 1.42 to 2.69), as seen in Table 3. Patients with diabetes had an adjusted OR of 1.46 (95%CI, 1.03 to 2.08) for de novo proteinuria.

**Table 3:** Multivariable association of de novo proteinuria

	Odds Ratio	95% CI	P Value
Age	1.00	0.99–1.01	0.65
Sex	0.79	0.58–1.08	0.14
Hispanic	0.96	0.63–1.46	0.86
Diabetes mellitus	**1.46**	1.03–2.08	0.03
Congestive heart failure	1.16	0.79–1.70	0.46
Coronary artery disease	0.61	0.40–0.94	0.03
Myocardial infarction	1.93	0.86–4.30	0.11
Cerebrovascular disease	1.04	0.68–1.60	0.85
Peripheral vascular disease	1.22	0.71 − 2.07	0.47
Liver disease	0.90	0.61–1.35	0.62
Dyslipidemia	0.83	0.57–1.20	0.32
Hypertension	1.37	0.95–1.99	0.09
AKI stage 2+	**1.95**	1.42–2.69	< 0.001

Model adjusted for age, sex, ethnicity, and presence of comorbidities

## Discussion

In this retrospective cohort of AKI survivors with known baseline kidney function, we found that approximately 60% of patients developed de novo proteinuria within one year after AKI discharge. We observed a dose-response relationship between AKI severity and subsequent proteinuria.

Prior studies assessing the development of CKD after AKI have mainly focused on eGFR evaluation. In fact, proteinuria, a marker of kidney damage and an important modifiable parameter, is hardly measured in AKI survivors post-discharge [15]. The mechanisms by which AKI leads to CKD have yet to fully elucidate [24] and the presence of shared risk factors for AKI, CKD, and cardiovascular disease leads to uncertainty about the real causal association between AKI and adverse outcomes. Establishing proteinuria as a complication of AKI is an important concept to incorporate into our understanding of AKI as a cause and accelerating factor for CKD development and progression.

In addition, focusing solely on eGFR to assess the sequela of AKI in overall kidney function underestimates the impact of episodes of AKI. Even if an AKI episode leads to no decrease in eGFR, the loss of total nephron mass can trigger a compensatory increase in single-nephron GFR and the hyperfiltration process [25]. Like in CKD, proteinuria after an episode of AKI can lead to inflammation and oxidative stress, contributing to kidney tissue damage and fibrosis.

Our study adds to the bidirectional association between AKI and proteinuria as a risk factor for AKI development and a sequela of AKI. Few studies have shown a high prevalence of proteinuria after an episode of severe AKI in adults [26, 27]. However, in most studies, it could not be determined if proteinuria was present before the AKI episode and was just a marker of increased risk for AKI development [28, 29]. Although our cohort is small, we only included patients with known kidney function and confirmed findings of a larger cohort [30]. In a larger study including two cohorts, the Assessment, Serial Evaluation, and Subsequent Sequelae of AKI (ASSESS-AKI) study and the subset of the Chronic Renal Insufficiency Cohort (CRIC), Hsu et al. were able to demonstrate that patients that developed AKI during hospitalization had a higher risk of subsequent worsening of proteinuria independently of other factors including diabetes [31].

Our study identified AKI based on administrative, diagnostic codes; however, we only included AKI patients with a prior baseline sCr and AKI based on the KDIGO criteria. We demonstrated that even in patients with stage 1 AKI, a high proportion of patients developed proteinuria. Furthermore, the risk of proteinuria was independently associated with the severity of AKI staging. These findings emphasized that the presence of more severe forms of AKI warrants rigorous attention to preventive care, screening, and follow-up.

The timing of proteinuria development is also an important finding in our study. As shown in Table 2, 59% of patients that developed proteinuria were already detected between within 3 months post-AKI.

In our cohort, we evaluated but did not emphasize other AKI-related complications like the development of de-novo CKD due to the small sample size. Further research should seek to find patients’ phenotypes pre-dating AKI to determine the relationships with downstream health outcomes. These phenotypes should seek to include social determinants of health, such as race/ethnicity, gender, and socioeconomic status, as well as behavioral, environmental, and material mediators, including physical and economic barriers to healthcare access, which would prevent patient screening post-AKI.

The main limitations of our study include the relatively small number of patients, precluding the analysis of the effect of more severe AKI and the degree of proteinuria. Because we did not have the ratio of urine albumin to urine total protein in all patients, we cannot further evaluate the progression of the severity of proteinuria. Our study is a single center, so our results may only be fully generalizable to some hospitalized patients, practice settings, or geographic areas. As an observational retrospective study, we cannot prove causal relationships between an episode of AKI and subsequent clinical outcomes, as we cannot rule out residual or unmeasured confounding.

In summary, we demonstrated that proteinuria is a frequent complication of AKI and that AKI is an independent risk factor for subsequent de novo proteinuria. Persistent proteinuria can be detected as early as three months after discharge, and more severe AKI is associated with a higher risk of proteinuria development. De novo proteinuria in AKI is a sign of kidney damage and can be a predictor of CKD progression and patient prognosis. Early detection and management are essential for improving outcomes and preventing long-term kidney damage. Further prospective studies are needed to determine whether strategies to detect patients at risk of worse kidney outcomes can delay the progression of CKD.

## Electronic supplementary material

Below is the link to the electronic supplementary material.

Supplementary Material 1

## Data Availability

The datasets used and analysed during the current study are available from the corresponding author on reasonable request.
