# Supplementary material for: Risk of de novo proteinuria following hospitalization with acute kidney injury
Source: BMC Nephrol. 2023 Jun 15;24:176. doi: 10.1186/s12882-023-03209-y (PMC10273748; doi:10.1186/s12882-023-03209-y)
Supplement: Supplementary file 1 — Supplementary Material 1 [file 12882_2023_3209_MOESM1_ESM.docx]

APPENDIX

Supplementary Tables

**Supplemental table 1. International Classification of Disease codes for comorbidities and outcomes.**

| Condition | Codes | Included Conditions |
| --- | --- | --- |
| Acute kidney injury (AKI) ^34-36^ | ICD-9: 580.0, 580.4, 580.8, 580.9, 584.0, 584.5, 584.6, 584.7, 584.9, 634.3, 635.3, 636.3, 638.3, 639.9  ICD-10: N00, N10, N17, O03.32, O03.82, O04.82, O07.32, O08.4, O90.4, T79.5 | Acute nephritic syndrome, acute tubulointerstitial nephritis, pregnancy AKI |
| Hypertension ^37-42^ | ICD-9: 401, 402, 403, 404, 405, 437.2  ICD-10: I10, I11, I12, I13, I15, I67.4 | Essential hypertension, hypertensive chronic kidney disease, hypertensive heart disease, hypertensive encephalopathy, secondary hypertension |
| Chronic kidney disease ^41,43-45^ | ICD-9: 250.40, 403, 404, 581, 582, 583, 585, 586, 587, 588, 753.12, 753.13  ICD-10: E10.2, E11.2, E13.2, E14.2, I12, I13, N01, N02, N03, N04, N05, N06, N07, N08, N11, N12, N14, N15, N16, N18, N19, N25, N26, Q61.1, Q61.2, Q61.3 | Hypertensive chronic kidney disease, diabetes with renal manifestations, chronic nephritic syndrome, chronic nephrotic syndrome, chronic glomerulonephritis, chronic tubulointerstitial nephritis, polycystic kidney disease |
| End stage renal disease (ESRD)^43,46-48^ | ICD9: 38.95, 39.27, 39.42, 39.43, 39.95, 54.98, 458.21, 585.6, 996.56, 996.68, 996.81, V42.0, V45.1, V56.0, V56.8  ICD10: N16.5, N18.6, T82.4, T86.1, Y60.2, Y61.2, Y62.2, Y84.1, Z48.22, Z49, Z94.0, Z99.2 | ESRD, dialysis, transplant and complications |
| Proteinuria ^41,49^ | ICD-9: 791.0, 581  ICD10: R80, N04, N06 | Nephrotic syndrome, isolated proteinuria |
| Diabetes ^38,42,46,50,51^ | ICD-10: E10, E11, E12, E13, E14 |  |
| Congestive Heart Failure ^43,44,51^ | I09.9, I11.0, I13.0, I13.2, I25.5, I42.0, I42.5–  I42.9, I43.x, I50.x, P29.0 |  |
| CAD ^38,42,50^ | I20, I121, I22, I24.0, I24.1, I24.8,  I24.9, I25.0, I25.10, I25.11,  I25.12, I25.13, I25.14, I25.19,  I25.2, I25.5, I25.6, I25.8, I25.9 |  |
| Myocardial infarction ^38,42,50^ | ICD10: I21.x, I22.x, I25.2 |  |
| Cerebrovascular disease ^38,42,50^ | G45.x, G46.x, H34.0, I60.x–I69.x |  |
| Peripheral vascular disease ^38,43,50,51^ | I70.x, I71.x, I73.1, I73.8, I73.9, I77.1, I79.0,  I79.2, K55.1, K55.8, K55.9, Z95.8, Z95.9 |  |
| Liver disease ^38,42,50^ | B18.x, I85.x, I86.4, I98.2, K70.x, K71.1, K71.3– K71.5, K71.7, K72.x– K74.x, K76.0, K76.2–K76.9, Z94.4 |  |
| Dyslipidemia ^52^ | E78 |  |
| Malignant neoplasm ^42^ | C000 – C97 |  |

**Supplemental table 2. De novo proteinuria by urinalysis**

| Pre-AKI |  | N |  |  |
| --- | --- | --- | --- | --- |
| Proteinuria-absent |  | 870 |  |  |
| Post-AKI |  |  |  |  |
| Proteinuria-eligible* |  | 759 |  |  |
| Proteinuria present** | | 472 |  |  |
| Post AKI UA results | 1+ | 202 |  |  |
|  | 2+ | 211 |  |  |
|  | 3+ | 57 |  |  |
|  | 4+ | 1 |  |  |
| Total patients with urinalysis results | | 471 |  |  |
| *Previously proteinuria-absent patients who had data available at post-AKI.  **Urinaylsis results were 1+ to 4+, or UPCR ≥0.3. | | | |  |
|  |  |  |  |  |
